# Osteonecrosis of femoral head after cement augmented TFNA nailing for an intertrochanteric fracture: A case report

**DOI:** 10.1016/j.tcr.2026.101305

**Published:** 2026-02-06

**Authors:** Srinivas Kasha, Ranjith Kumar Yalamanchili, Anurag Gurram, Sindhura Yamajala, G.P.R.K Rohit

**Affiliations:** a Department of Orthopaedics, Krishna Institute of Medical Sciences, Minister Road, Secunderabad, Telangana, India; b Department of Orthopaedics, All India Institute of Medical Sciences, Bibinagar, Telangana, India

**Keywords:** ONFH in TFNA, ONFH with bone cement augmentation, Bone cement augmentation, Bone cement augmented TFNA, Bone cement augmentation in intertrochanteric fractures, ONFH

## Abstract

Failure of fixation devices (cut through) in proximal femoral fractures continues to be a concern in severe osteoporosis. Bone cement augmentation using TFNA (Trochanteric Femoral Nail-Advanced™; DePuy Synthes) increases the bone implant interphase and thereby the biomechanical stability, offering better resistance to avoid cut through. Cement augmentation does not improve implant positioning or reduction but only provides mechanical advantage to the osteoporotic bone and was reported as a safer technique in literature. Most commonly reported complication following cement augmented TFN-A insertion is cement leakage through the fracture. While ONFH (Osteonecrosis of Femoral Head) following Intertrochanteric fractures is not an uncommon complication, its incidence in AO 31A3.3 fracture pattern is very rare. An in vitro study demonstrated that there was a transient increase in intraosseous pressure with injection of 6 ml of bone cement, yet posing a very low risk of developing ONFH. Despite appropriate primary fixation for an AO 31 A3.3 fracture using a TFN-A, we observed ONFH following fracture healing. This subsequently progressed to cephalic screw cut-through and the development of an acetabular bone defect, necessitating total hip arthroplasty with acetabular augments. Among patients treated with cement augmented TFN-A, any new complaint of hip pain after fracture healing should prompt strong suspicion for ONFH and warrant thorough evaluation. Undue delay will progress ONFH and cause structural damage to acetabulum due to the exposed helical blade.

## Introduction

Unlike with an intracapsular fractures of neck of femur, Osteonecrosis of Femoral head (ONFH) is an uncommon complication following an Intertrochanteric (IT) fracture. ONFH following intertrochanteric fractures was first reported by Picchio et al. in 1954 [1]. Barquet et al. reported that the incidence of ONFH after fixation of intertrochanteric fractures as 1.37% in the first two years after injury [2]. Chen et al. reported seven cases of ONFH in unstable fractures treated by Gamma nail and most of them in the series had fracture geometry extending proximally or with severe comminution [3]. Failure of fixation devices (cut through) in proximal femoral fractures continues to be a concern, particularly among patients with osteoporosis. Bone cement augmentation using TFNA (Trochanteric Femoral Nail - Advanced™; DePuy Synthes) improves the bone-implant interphase and biomechanical stability in osteoporotic patients [4]. Cement augmentation does not improve implant positioning or reduction but only provides a mechanical advantage of anchorage in osteoporotic femoral head and was reported as a safer technique in literature. Apart from risk of leakage of bone cement into the joint and intraoperative physiological changes in heart rate and blood pressure during bone cement injection, no case of ONFH following TFNA insertion for primary fixation of intertrochanteric fractures has been reported in literature [5], [6]. We report a case of ONFH following treatment of a reverse oblique intertrochanteric fracture using a TFNA nail.

## Case report

**Fig. 1:**
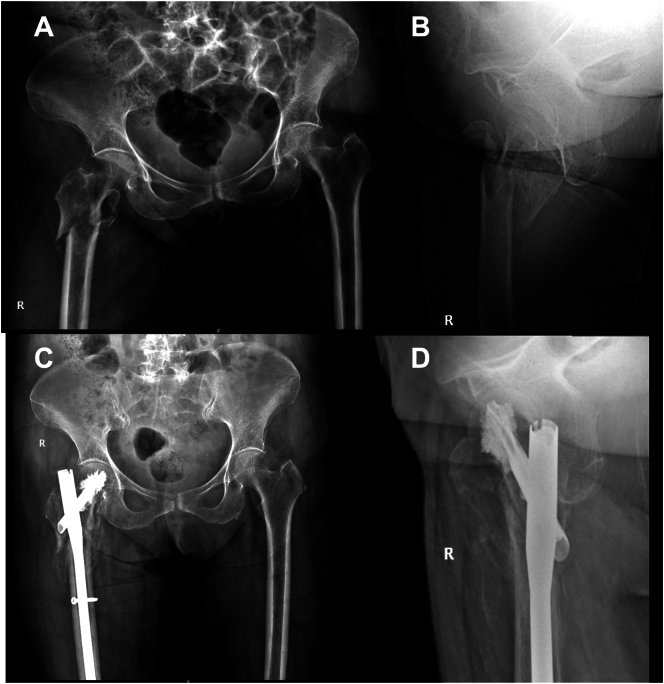

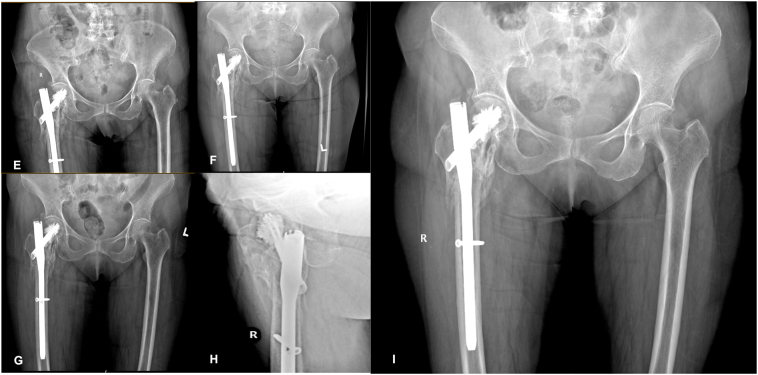
A–D): A) Pre op radiograph AP view and B) lateral view showing IT fracture. C: Post-operative radiograph at 6 weeks AP view and D) lateral view of TFNA fixation of fracture. E–I: E) Post-operative radiograph at 16 weeks, F) at 7 months follow up, G&H) Ap and lateral view radiograph at 11 months follow up and I) AP view at 15 months follow up.

**Fig. 2:**
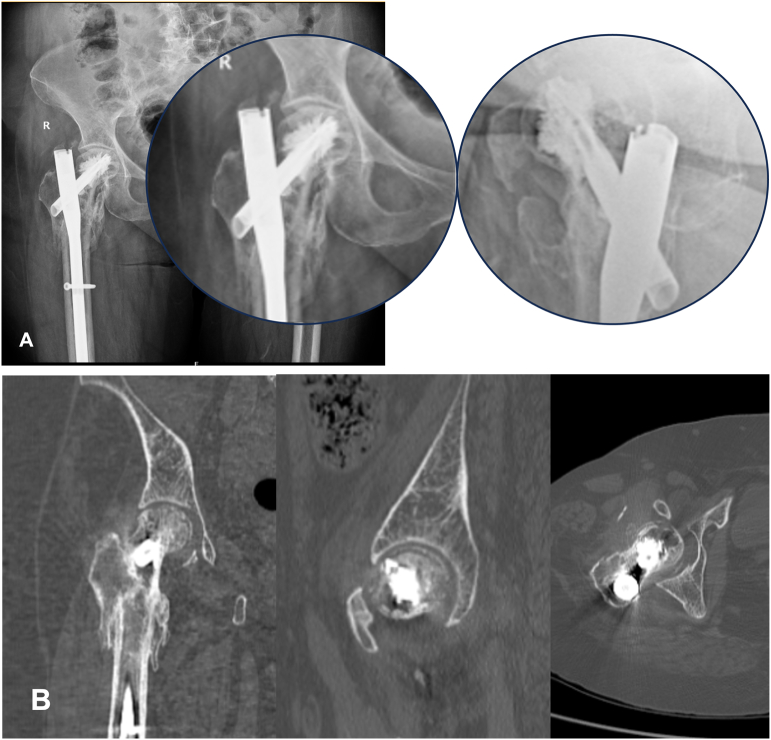
A) 18th month Post op x ray AP and lateral View showing Changes of ONFH– subchondral bone collapse and subchondral cysts. b): CT scan hip showing Changes of ONFH with crescent sign.

**Fig. 3:**
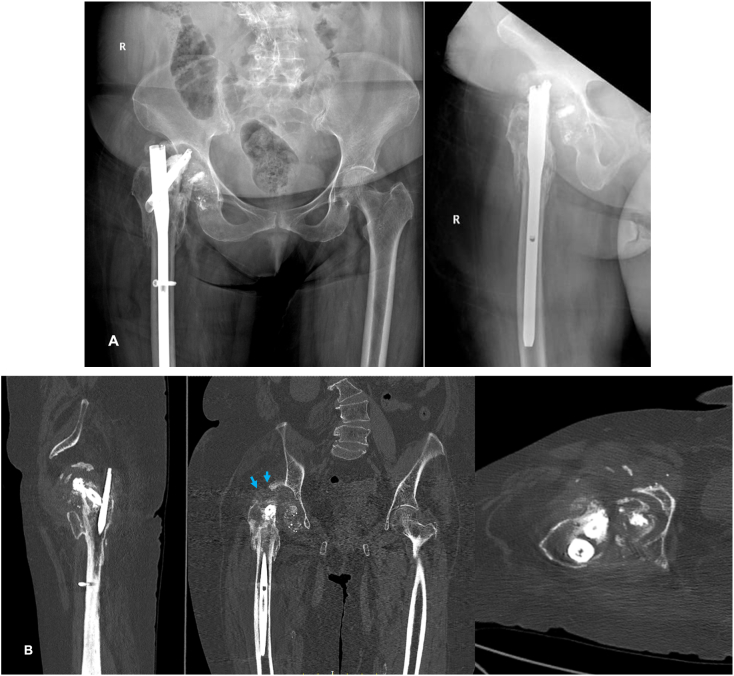
A) Cut through of helical blade – X ray hip Ap and lateral views at 23rd month follow up visit. B) CT Sections Showing ONFH, Cut Through of femoral blade and acetabular erosions (Blue arrow).

**Fig. 4:**
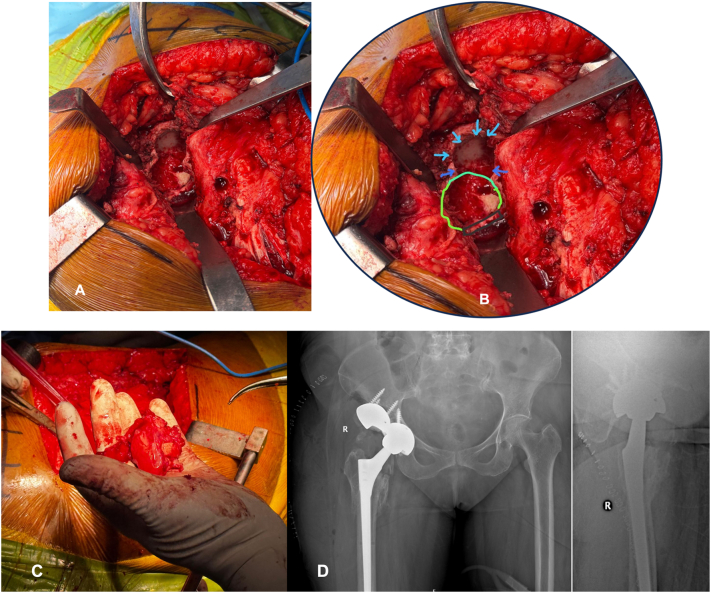
A): Clinical Intra operative image showing acetabular erosions. B) Close up view with markings – Blue arrow mark pointing to the acetabular erosion by cephalic screw, green depicts acetabular margin, black line – Transverse Acetabular ligament (TAL). C: Femoral head with complete delamination of cartilage and sclerotic. D: Post op radiograph AP and Lateral view showing total Hip arthroplasty with acetabular augments.

A 85 year old female patient (Body Mass Index ∼23.2) with a known history of Hypertension, Type II Diabetes and Cerebrovascular accident 10 years ago sustained a reverse oblique intertrochanteric fracture (AO31A3.3). Her pre-injury ambulatory status was independent, although limited to domestic activities. She was managed with closed reduction and internal fixation using TFNA (DePuy Synthes, Raynham, Massachusetts, USA). Closed reduction coupled with indirect reduction techniques using hoffman retractor to reduce anterior spike was used intraoperatively. Following satisfactory reduction, TFNA was introduced and augmented with a 3 ml of Traumacem™ V (Poly Methyl Metha Acrylate - DePuy Synthes, Raynham, Massachusetts, USA), that was injected in incremental dosages of 2 ml and 1 ml through the helical blade. There was no intraoperative leakage of cement noted [Fig. 1]. Post operatively, patient was mobilized with walker and in 6 weeks, patient was mobilized with full weight bearing. At 16 weeks, patient resumed to pre injury status and could carry out all her routine daily activities without any support. She was followed every third month after bony union for clinico-radiological assessment and was treated for osteoporosis with Teriparatide injections by subcutaneous route for six months. At 18 months of follow up, radiograph showed ONFH and the patient was evaluated with a CT (Computerised Tomography) scan of hip, which revealed crescent sign [Fig. 2]. Patient did not have any limitations in function or pain and continued to do all her routine activities and was advised regular evaluation to look for progression of ONFH. At 23rd month of post-operative follow up; she presented with complaints of painful hip and disability lasting from 6 weeks and upon evaluation it was noted that ONFH progressed with cut through of helical blade causing a significant defect in acetabulum [Fig. 3]. She has no history of corticosteroid use or any other known aetiological factors associated with ONFH. She underwent implant removal and a total hip arthroplasty (THA) and intraoperatively it was noted that there was a significant acetabular defect that needed augments and the femoral head was necrotic with complete delamination of articular cartilage [Fig. 4]. Post THA, patient is independently ambulating without any functional limitation.

## Discussion

Local factors causing ONFH in per-trochanteric fractures apart from fracture geometry can be extraosseous disruption [7], [8] or local thermal necrosis of femoral head [2], [3], [4], [5]. Complications following intertrochanteric fracture fixation are primarily related to mechanical factors and poor bone quality that leads to superior cutout, pull out of screw or blade in the cephalic component [5]. Even when surgeon controlled variables like fracture reduction and cephalic screw placement are optimized, Osteoporosis significantly influences the outcome of treatment of IT fractures as a crucial extrinsic determinant. Cement augmentation in TFNA fixation improves the biomechanical stability in osteoporotic intertrochanteric fractures and addresses the challenges posed by poor bone stock, thereby reducing the risk of implant related complications. In cases of revision fixation following failure of primary fixation(‘Z’ effect, reverse ‘Z’ effect, early varus collapse, cut through of screw in head of femur), augmented TFNA is an effective alternative to traditional arthroplasty [9].

Complications following bone cement augmented TFNA reported in literature include cement leakage into the joint, cement interposition at the fracture site, bone cement implantation syndrome (BCIS),Pulmonary embolism (PE), ONFH and cartilage damage [7], [10].

Schuetze et al. in their largest retrospective series of 152 patients operated with TFNA augmented with Traumacem™ V, observed minor physiological reaction (changes in heart rate, oxygen saturation and blood pressure requiring vasoactive drug intervention) within the first five minutes of cement insertion in 57 patients [6]. Agarwala et al. reported a case of varus failure with helical blade cut out requiring a revision surgery [11]. They attributed the failure due to an increased Tip apex distance (TAD) of cephalic screw of about 29 mm and negative variance in an osteoportic bone, without any evidence of ONFH.

Aguado et al. in their retrospective cohort study of 30 cement-augmented cases, reported that one patient developed osteonecrosis of the femoral head (ONFH) and subsequently required total hip arthroplasty (THA) at 12 months [5]. Notably, this patient had a prior history of contralateral THA due to ONFH, suggesting a possible individual predisposition to the condition. Huyke-Hernandez et al. reported a case of osteonecrosis of the femoral head with cut-through following revision surgery using augmented TFNA for a previously failed fixation [9]. The occurrence of ONFH and cut-through in this case may be attributed to the revision procedure itself and the pre-existing bone defects resulting from the initial implant failure. Most of the other studies that reported revision surgeries using augmented TFNA did not document any cases of ONFH [9].

The application of bone cement in vertebroplasty and kyphoplasty is widely practiced, with reported possible complications related to cement leakage [12]. There are no reports of Osteonecrosis of vertebral bodies, despite using similar volume of bone cement perhaps due to rich vasculature of vertebrae. Blankstein et al. in their in vivo model, demonstrated that the injection of bone cement results in a transient increase in intraosseous pressure within the femoral head, potentially having a chance leading to ONFH, particularly when injected rapidly and when volumes exceed 6 ml [13]. However, the rapid applicability of bone cement to clinical fracture scenarios is limited as the injecting a cement encounters high resistance, which is primarily due to the constrained distribution of the cement cloud within the femoral head; rather than due to elevated intraosseous pressure. In their study, they did not consider the effect of exothermic reaction generated due to setting of the cement and causing local tissue necrosis. Future studies should directly evaluate intraosseous pressure and temperature changes during cement augmentation to clarify their clinical role in the development of osteonecrosis of the femoral head.

## Conclusion

While prior studies have largely linked ONFH following intertrochanteric fractures to fracture geometry or revision procedures, the present case highlights the potential role of bone cement augmentation, possibly mediated by increased intraosseous pressure or the exothermic reaction during cement polymerization. This underscores the importance of continued clinical monitoring, particularly in patients presenting with hip pain even after fracture healing following TFNA implantation. Early recognition of ONFH is crucial to prevent significant structural bone loss that can result due to erosion caused by the rigid helical blade in TFNA.

## Ethics statements

Patient whose case is reported has given an informed consent to submit the relevant radiographs and intraoperative findings of the patient for publication.

## CRediT authorship contribution statement

**Srinivas Kasha:** Visualization, Validation, Supervision, Software, Resources, Project administration, Methodology, Investigation, Funding acquisition, Formal analysis, Data curation, Conceptualization. **Ranjith Kumar Yalamanchili:** Writing – original draft, Visualization, Validation, Software, Resources, Methodology, Investigation, Formal analysis, Data curation, Conceptualization. **Anurag Gurram:** Writing – review & editing, Visualization, Validation, Supervision, Software, Resources, Project administration, Methodology, Investigation, Formal analysis, Data curation. **Sindhura Yamajala:** Writing – review & editing, Visualization, Validation, Software, Resources, Methodology, Investigation, Formal analysis, Conceptualization. **G.P.R.K. Rohith:** Writing – review & editing, Validation, Supervision, Software, Resources, Project administration, Investigation, Funding acquisition, Formal analysis, Data curation.

## Informed consent

The patient was informed that data concerning the case would be submitted for publication, and she provided consent.

## Declaration of Generative AI and AI-assisted technologies in the writing process

Authors declare that there is no use of generative AI in the manuscript preparation process.

## Funding

There was no Funding Involved in the treatment and reporting of the case.

## Declaration of competing interest

The authors declare that they have no known competing financial interests or personal relationships that could have appeared to influence the work reported in this paper.
